# Effect of remote ischemic preconditioning on postoperative acute kidney injury among patients undergoing cardiac and vascular interventions: a meta-analysis

**DOI:** 10.1007/s40620-016-0301-x

**Published:** 2016-04-18

**Authors:** Bingjue Li, Xiabing Lang, Luxi Cao, Yuchen Wang, Yingying Lu, Shi Feng, Yi Yang, Jianghua Chen, Hong Jiang

**Affiliations:** 1 Kidney Disease Center, The First Affiliated Hospital, School of Medicine, Zhejiang University, Qinchun Road 79#, Hangzhou, 310003 People’s Republic of China; 2 Kidney Disease Immunology Laboratory, The Third Grade Laboratory, State Administration of Traditional Chinese Medicine of P.R. China, Hangzhou, People’s Republic of China; 3 Key Laboratory of Multiple Organ Transplantation, Ministry of Health, Hangzhou, People’s Republic of China; 4 Key Laboratory of Nephropathy, Zhejiang Province, Hangzhou, Zhejiang People’s Republic of China

**Keywords:** Remote ischemic preconditioning, Acute kidney injury, Cardiac and vascular interventions, Meta-analysis

## Abstract

It is currently controversial whether remote ischemic preconditioning (RIPC) reduces the incidence of acute kidney injury (AKI) in patients undergoing cardiovascular interventions. The main objective of this meta-analysis was to investigate whether RIPC provides renal protection for patients undergoing cardiac or vascular surgery. We searched the PubMed database (1966-Oct 2015), Embase database (1966-Oct 2015), Google Scholar, Cochrane Library, ClinicalTrials Database and Open Grey. Then we conducted a meta-analysis of the randomized controlled trials that met the inclusion criteria of our study. The interventions included use of an inflatable tourniquet around the limbs or cross-clamping of the iliac arteries before surgery (RIPC groups) and general cardiovascular intervention (control groups). The main outcomes examined included the incidence of AKI; changes in acute kidney injury biomarkers; and use of renal replacement therapy. Other outcomes examined included in-hospital mortality and the lengths of hospital stay and intensive care unit (ICU) stay. Finally, we screened 26 eligible studies containing 6699 patients who underwent cardiac or vascular interventions with RIPC (n = 3343) or without RIPC (n = 3356). The AKI incidence was decreased in the RIPC group as was the length of ICU stay. There were no differences in the changes in AKI biomarkers, use of renal replacement therapy or in-hospital mortality between the two groups. Remote ischemic preconditioning may decrease the occurrence of AKI in cardiovascular surgery patients. Since studies included have a significant heterogeneity, meta-analyses using a stricter inclusion criteria are needed to clarify the renoprotection effect of RIPC.

## Introduction

Acute kidney injury (AKI) is a serious post-operation complication in cardiac surgery patients [1]; its incidence ranges from 3 to 42 % [2–10], and 1 to 5 % of AKI patients require dialysis therapy [1, 2, 4, 9, 11]. The mortality of AKI patients has been reported to be as high as 40–80 % [1, 7, 9]. Although this clinical problem is gaining increased attention, there are still no efficient methods to prevent AKI after cardiac and vascular interventions [5, 6, 12–14]. A double-blinded, placebo-controlled multicenter study conducted by Julier et al. [15] confirmed that sevoflurane preconditioning reduces the increase in postoperative plasma cystatin C (Cys C) concentration; however, that trial did not investigate the relationship between sevoflurane preconditioning and perioperative AKI prevalence.

Remote ischemic preconditioning (RIPC) consists of cycles of transient non-fatal ischemia in one tissue to enhance the toleration of a subsequent prolonged fatal ischemia in distant organs [16]. The protective mechanism of RIPC to specific organs has been illustrated by several studies [14, 17, 18]; however, whether remote ischemic preconditioning has a positive clinical effect on renal function in cardiac and vascular surgery patients remains unclear. Some previous randomized controlled trials (RCTs) showed that RIPC reduces AKI incidence in cardiac surgery patients, but other studies had conflicting results. Several previous systematic reviews also demonstrated controversial results [19–23]. In recent years, more relevant RCTs have been carried out and published, so we performed a meta-analysis to verify the effect of RIPC on acute kidney injury in patients undergoing cardiovascular interventions.

## Methods

### Study design

Studies that met the following inclusion criteria were included in this meta-analysis: (1) RCT design; (2) study participants underwent some type of elective or acute cardiac or vascular surgery; (3) RIPC intervention, regardless of the duration or number of cycles; vessel occlusion models were also included; the control group intervention was standard treatment without RIPC or with sham RIPC; and (4) report of the incidence of AKI.

The primary outcome analyzed was the incidence of AKI. The secondary outcome measures included change in renal biomarkers after surgery, the use of renal replacement therapy, in-hospital mortality, the length of hospital stay and the length of intensive care unit (ICU) stay.

### Search strategy

A literature search was conducted after establishing the inclusion criteria. We searched published articles in the PubMed (1966-Oct 2015), Embase (1966-Oct 2015), Google Scholar and Cochrane Library databases. We also searched ClinicalTrials.gov and Open Grey for unpublished and ongoing trials. There were no language or region restrictions. The following Medical Subject Heading terms and text words were used: ischemic preconditioning, cardiovascular surgical procedures, randomized controlled trial, controlled clinical trial, remote ischemic preconditioning. Titles and abstracts were screened by two authors (L-xC, Y-cW) to guarantee their concordance with the inclusion criteria. Full text screening was conducted by the same two persons after preliminary screening if the article’s eligibility could not be determined by screening the title and abstract.

### Data extraction

Data extraction was performed by another two authors (Y-yL, SF) using a standardized data extraction form. Disagreements were resolved by a third person who served as an intermediary (B-jL) and made the final decision. Every trial was carefully assessed, and the following data were extracted: patient demographic characteristics, types of cardiovascular interventions, RIPC protocol, dose of contrast medium, AKI definition, incidence of AKI, in-hospital mortality, length of hospital stay, length of ICU stay, incidence of kidney replacement therapy, serum or plasma creatinine levels before and 24 and 48 h after surgery, and glomerular filtration rates (GFRs) at 24 and 48 h after surgery.

### Study validity assessment

The Jadad scale was used to evaluate the methodological quality of the eligible trials. This scoring standard examines randomization, blinding, and explanation for withdrawals and dropouts [24, 25]. The modified Jadad scale, which includes the additional factor of item allocation concealment, was also used, with a score of 1–3 indicating low quality and a score of 4–7 indicating high quality [26]. Intention-to-treat (ITT) was also analyzed. We also judged risk of bias for each included study in terms of selection bias, performance bias, detection bias and attrition bias.

### Statistical analysis

We mainly utilized the software Review Manager (RevMan) version 5.3 to analyze the extracted data (Copenhagen: The Nordic Cochrane Centre, The Cochrane Collaboration, 2014). Risk ratios (RRs) with 95 % confidence intervals (CIs) were calculated for dichotomous outcomes, and mean differences with 95 % CI were calculated for continuous outcomes. Statistical heterogeneity was assessed using the χ^2^ test, and we determined the percentage of total variation across studies using the Higgins I^2^ statistic. We compared our primary analysis with random-effects models using the Knapp–Hartung method to determine the robustness of the pooled effects.

## Results

### Search results

**Fig. 1:**
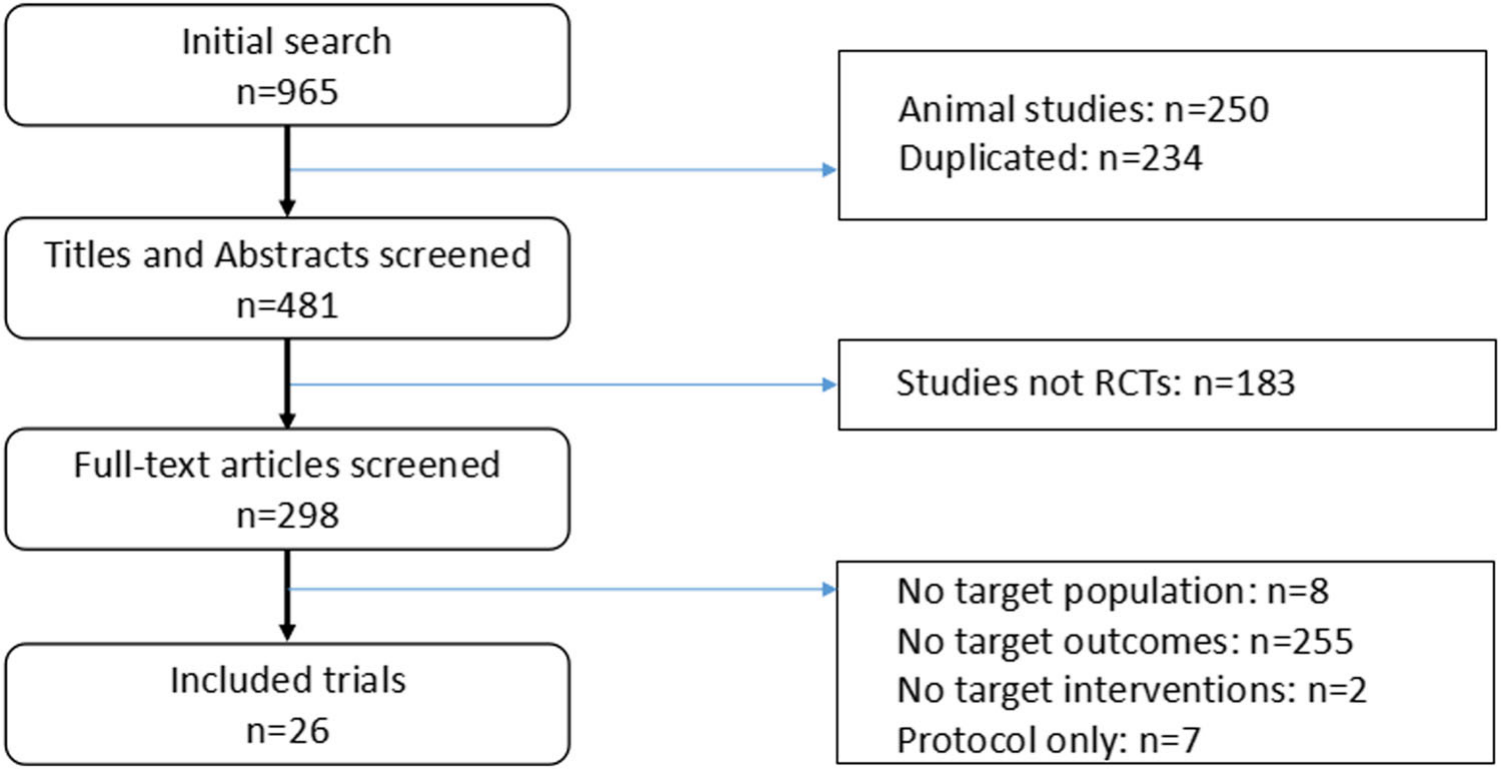
Flowchart of article selection. *RCT* randomized controlled trial

The search initially identified 965 articles; 234 duplicated articles and 250 animal studies were excluded. After title and abstract screening, 183 nonrandomized trials were excluded. Then, by full-text assessment of the remaining articles, we finally identified 26 eligible randomized controlled trials [14, 16, 27–50] (excluded articles: no target population: n = 8; no target outcomes: n = 255; no target interventions: n = 2; protocol only: n = 7, Fig. 1).

### Study characteristics

A total of 6699 patients were enrolled in the 26 included studies, with 3343 patients randomized to the RIPC group and 3356 randomized to the control group. Sixteen studies examined cardiac surgery [14, 16, 28, 29, 31–33, 36, 39, 40, 43, 46–50], six studies examined percutaneous coronary intervention [30, 34, 35, 37, 41, 45], and four studies examined vascular surgery [27, 38, 42, 44]. The RIPC protocols were different between studies: 24 studies used an inflatable tourniquet around the limbs [14, 16, 27–50], and two studies used cross-clamping of the iliac arteries [27, 44]. The participants in one of the studies were children [39], while those of all other studies were adults. Seven studies applied contrast medium [30, 34, 35, 37, 41, 42, 45]. The key characteristics of the included studies are shown in Table 1.

**Table 1:** Demographic data of included trials

References	N. Patients^a^	Mean age (year)^a^	Males (%)^a^	HTN^a^	DM^a^	Baseline Scr (μmol/l)^a^	Surgical procedure	Contrast (ml)	RIPC procedure	AKI definition
Ali et al. [27]	41/41	74/75	93/93	21/26	2/2	102/101	Elective open AAA repair	None	Cross-clamping of iliac arteries	Peak Scr >177 mmol/l
Candilio et al. [28]	89/89	65/66	81/75	65/67	28/24	NA	CABG, valve surgery	None	Inflatable tourniquet around the limbs	Scr >26.4 mmol/l or 150–200 % increase from baseline and/or urine output <0.5 ml/kg/h for >6 h
Choi et al. [29]	38/38	57/60	38/38	8/10	1/4	80.4/81.3	CABG, valve surgery, Bentall procedure	None	Inflatable tourniquet around the limbs	AKIN criterion
Er et al. [30]	50/50	73.2/72.7	68/74	45/46	32/32	144.1/143.2	Coronary angiography	124/103	Inflatable tourniquet around the limbs	Scr ≥25 % or ≥0.5 mg/dl increase from baseline
Gallagher et al. [31]	43/43	68.7/72.8	76.7/83.7	34/37	27/28	121.1/121.1	CABG, CABG + AVR	None	Inflatable tourniquet around the limbs	Scr >0.3 mg/dl increase from baseline within 48 h of surgery
Hausenloy et al. [48]	801/822	76.1/76.3	556/586	602/599	203/211	NA	On-pump CABG	None	Inflatable tourniquet around the limbs	KDIGO criteria
Hong et al. [32]	35/35	64.5/64.8	80/66	23/25	12/13	NA	OPCAB	None	Inflatable tourniquet around the limbs	Scr >2.0 mg/dl and Scr >0.7 mg/dl increase from baseline
Hong et al. [33]	644/646	60.8/60.9	61.3/61.3	325/297	183/204	NA	CABG, valve surgery	None	Inflatable tourniquet around the limbs	Scr of 2.0 mg/dl and Scr ≥0.7 mg/dl increase from baseline
Hoole et al. [34]	104/98	63.2/61.8	81/76	53/51	24/20	NA	Elective PCI	196.7/187.5	Inflatable tourniquet around the limbs	Scr >25 % increase from baseline
Igarashi et al. [35]	30/30	71.3/70.8	66.7/76.7	NA	NA	101.7/99.0	PCI	92.9/91.8	Inflatable tourniquet around the limbs	L-FABP >17.4 μg/g Cr or >25 % increase from baseline within 24 h after use of CM
Kim et al. [36]	27/27	58/57	59.3/51.9	10/8	5/2	NA	CABG, valve surgery, Bentall operation	None	Inflatable tourniquet around the limbs	Scr >50 % or >0.3 mg/dl increase from baseline within 48 h after surgery
Luo et al. [37]	101/104	59.2/59.3	77.2/75	19/20	26/31	NA	Elective PCI	154/145	Inflatable tourniquet around the limbs	Scr >25 % increase from baseline
Meybohm et al. [16]	90/90	70/68	69/77	79/73	21/17	72.5/77.8	CABP, valve surgery, distal anastomoses	None	Inflatable tourniquet around the limbs	AKIN criteria
Meybohm et al. [49]	692/693	65.8/66.0	508/520	573/573	166/178	NA	Elective CABG	None	Inflatable tourniquet around the limbs	Scr ≥2 fold from baseline
Murphy et al. [38]	31/31	75/69	94/77	20/16	7/5	86/90	Elective AAA repair	None	Inflatable tourniquet around the limbs	AKIN criteria
Pedersen et al. [39]	54/51	1.0/0.9	46/65	NA	NA	35/32	Operation for complex CHD	None	Inflatable tourniquet around the limbs	RIFLE criteria
Pinaud et al. [50]	50/49	75.8/72.9	27/24	37/40	6/8	NA	Aortic valve surgery	None	Inflatable tourniquet around the limbs	AKIN criteria
Rahman et al. [40]	80/82	63/65	89/88	44/52	3/0	98.1/96.4	CABG	None	Inflatable tourniquet around the limbs	Scr >0.5 mg/dl increase from baseline
Savaj et al. [41]	48/48	63.0/60.9	35.4/29.2	32/36	48/48	114.9/97.2	Coronary angiography	126.6/123.8	Inflatable tourniquet around the limbs	KDIGO criteria
Venugopal et al. [43]	38/40	64/66	30/34	29/22	0/0	84.58/84.24	Elective CABG	None	Inflatable tourniquet around the limbs	AKIN criteria
Walsh et al. [42]	18/22	74/76	100/100	8/12	3/2	95/94	Elective endovascular aneurysm repair	309/286	Inflatable tourniquet around the limbs	Decrease in eGFR ≥20 % from baseline
Walsh et al. [44]	22/18	75/72	72.7/100	12/16	1/0	97/88	Elective open AAA repair	None	Cross-clamping of iliac arteries	Decrease in eGFR ≥20 % from baseline
Yamanaka et al. [45]	47/47	67/67	76/76	29/31	14/17	72.5/76.9	Emergency PCI	177/199	Inflatable tourniquet around the limbs	Scr >0.5 mg/dl or >25 % increase from baseline 48–72 h after use of CM
Young et al. [46]	48/48	65.5/64.4	60.4/64.6	NA	NA	102/95	CABG, valve surgery	None	Inflatable tourniquet around the limbs	RIFLE criterion
Zarbock et al. [14]	120/120	70.1/70.6	63.3/62.5	116/116	46/44	97.24/106.08	CABG, valve surgery	None	Inflatable tourniquet around the limbs	KDIGO criteria
Zimmerman et al. [47]	59/59	62/65	69/68	44/50	24/21	82.2/84.0	CABG, valve surgery	None	Inflatable tourniquet around the limbs	AKIN criterion

*AAA* abdominal aortic aneurysm, *AKI* acute kidney injury, *AKIN* Acute Kidney Injury Network, *HTN* hypertension, *DM* diabetes mellitus, *CABG* coronary artery bypass grafting, *CHD* congenital heart disease, *eGFR* estimated glomerular filtration rate, *NA* not available, *PCI* percutaneous coronary intervention, *RIFLE* risk, injury, failure, loss, and end-stage disease, *RIPC* remote ischemic preconditioning, *Scr* serum creatinine, *CM* contrast medium

^a^RIPC group/control group

### Quality assessment

**Fig. 2:**
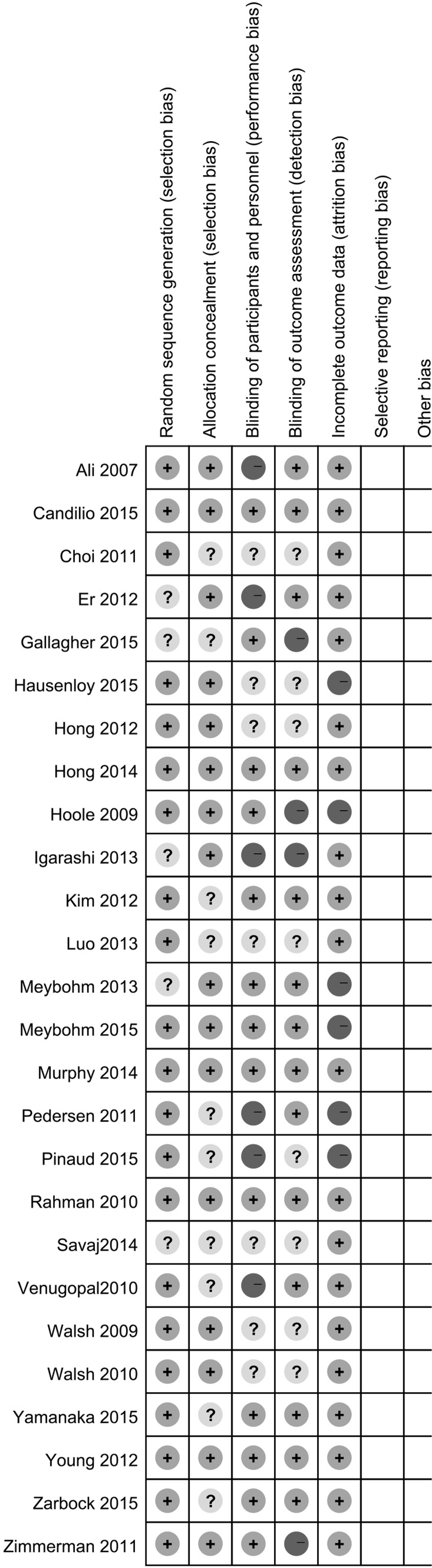
Risk of bias table: *green* low risk of bias, *yellow* unclear risk of bias, *red* high risk of bias (color figure online)

Two authors (YY, X-bL) independently assessed the quality of the 26 studies using the Jadad scale and the modified Jadad scale. Twenty-one (80 %) trials [14, 16, 27–29, 32–34, 36–40, 42–45, 48–50] had a relatively high methodological quality based on the Jadad scale, while 19 (73 %) trials [14, 16, 27, 28, 30, 32–36, 38, 40, 42, 44, 45, 47–49] had a relatively high methodological quality based on the modified Jadad scale. The randomization methods were adequate in 20 studies. Allocation concealment was adequate in 16 studies. Blinding was adequate in ten studies (Fig. 2). All studies had clear explanations for withdrawals and dropouts. Only six studies did not meet the ITT analysis criteria. The details of the quality assessment are shown in Table 2.

**Table 2:** Quality assessment of included trials

References	Randomization method	Allocation concealment	Blinding	Explanation for withdrawals/dropouts	Intention-to-treat analysis	Jadad Score	Modified Jadad Score
Ali et al. [27]	Computer-generated random list	Sealed envelopes	Single blind	Yes	Yes	3	5
Candilio et al. [28]	Computer-generated random list	Sealed envelopes	Double blind	Yes	Yes	5	7
Choi et al. [29]	Computerized randomization table	Unclear	Unclear	Yes	Yes	3	3
Er et al. [30]	Unclear	Sealed envelopes	Single blind	Yes	Yes	2	4
Gallagher et al. [31]	Unclear	Unclear	Single blind	Yes	Yes	2	2
Hausenloy et al. [48]	By means of a secure website	Sealed envelopes	Unclear	Yes	No	3	5
Hong et al. [32]	Computer-generated random list	Sealed envelopes	Unclear	Yes	Yes	3	5
Hong et al. [33]	Computer-generated random list	Sealed envelopes	Double blind	Yes	Yes	5	7
Hoole et al. [34]	Computer-generated randomization procedure	Sealed envelopes	Single blind	Yes	No	3	5
Igarashi et al. [35]	Unclear	Sealed envelopes	Non-blind	Yes	Yes	2	4
Kim et al. [36]	Computer-generated random list	Unclear	Double blind	Yes	Yes	5	5
Luo et al. [37]	Simple digital method of randomization	Unclear	Unclear	Yes	Yes	3	3
Meybohm et al. [16]	Unclear	Sealed envelopes	Double blind	Yes	No	4	6
Meybohm et al. [49]	Performed by the Clinical Trial Centre Leipzig	Sealed envelopes	Double blind	Yes	No	5	7
Murphy et al. [38]	Computer-generated random list	Sealed envelopes	Double blind	Yes	Yes	5	7
Pedersen et al. [39]	Computerized randomization table	Unclear	Single blind	Yes	No	3	3
Pinaud et al. [50]	Computerized randomization table	Unclear	Single blind	Yes	No	3	3
Rahman et al. [40]	Computer-generated randomization procedure	Sealed envelopes	Double blind	Yes	Yes	5	7
Savaj et al. [41]	Unclear	Unclear	Unclear	Yes	Yes	2	2
Venugopal et al. [43]	Computer-generated random list	Unclear	Single blind	Yes	Yes	3	3
Walsh et al. [42]	Computer-generated random list	Sealed envelopes	Unclear	Yes	Yes	3	5
Walsh et al. [44]	Computer-generated random list	Sealed envelopes	Unclear	Yes	Yes	3	5
Yamanaka et al. [45]	Computer-generated random list	Unclear	Double blind	Yes	Yes	5	5
Young et al. [46]	Online randomization sequence generator	Sealed envelopes	Double blind	Yes	Yes	5	7
Zarbock et al. [14]	Computer-generated random list	Unclear	Double blind	Yes	Yes	5	5
Zimmerman et al. [47]	Block randomization generated by study coordinator	Sealed envelopes	Single blind	Yes	Yes	2	4

### Outcomes

#### Incidence of AKI

**Fig. 3:**
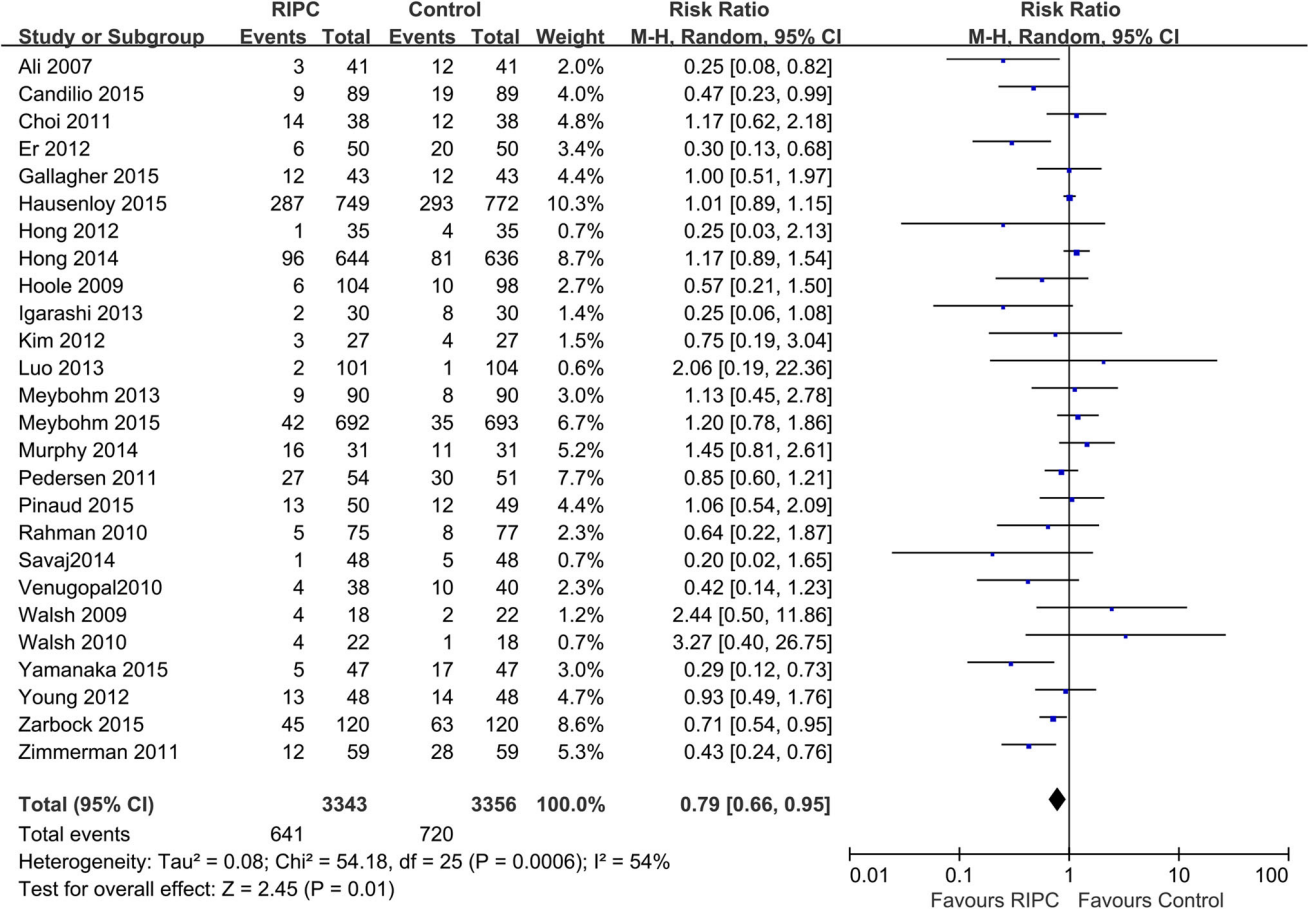
Meta-analysis of AKI incidence between RIPC and control groups. *CI* confidence interval, *KH* Knapp–Hartung method

**Fig. 4:**
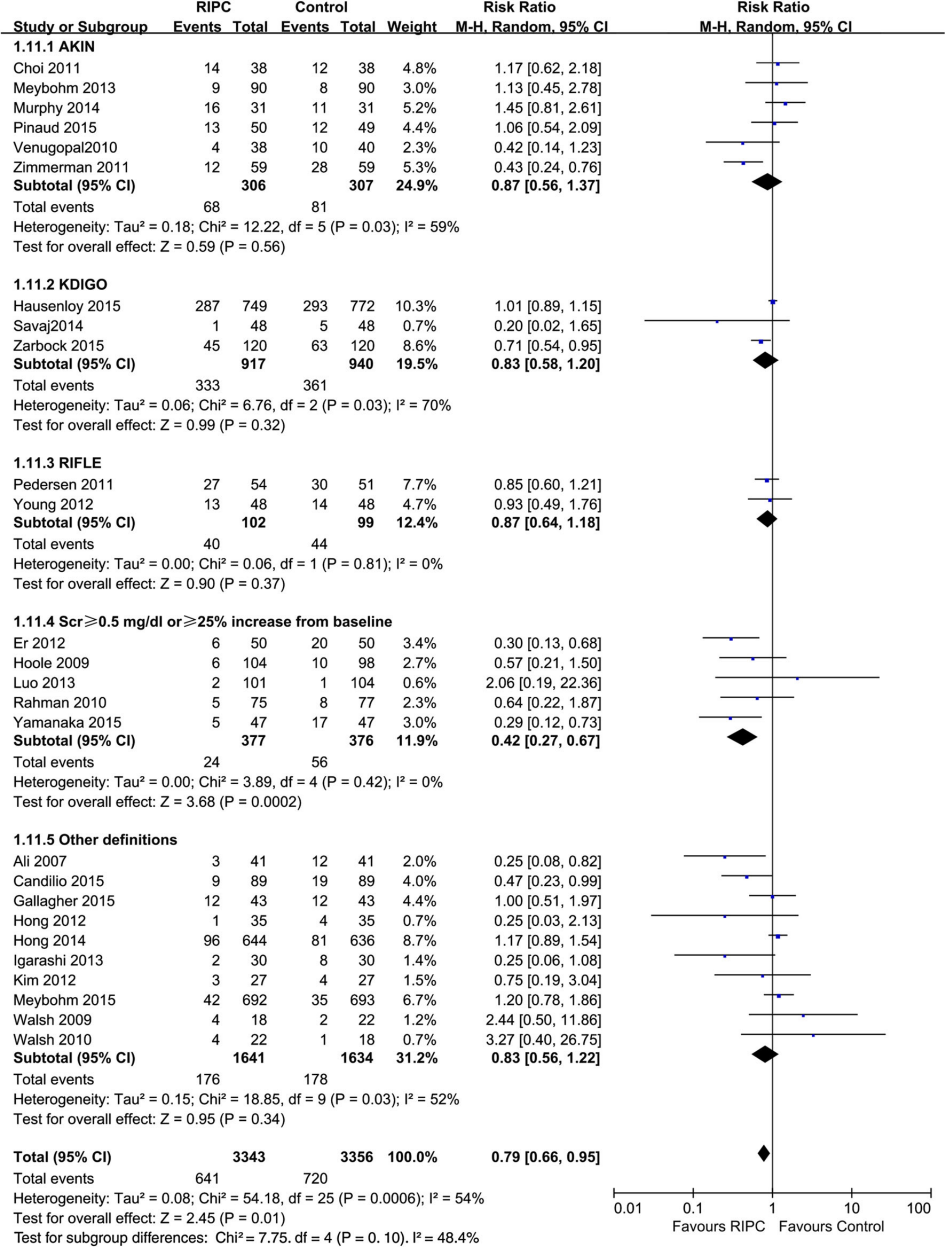
Subgroup analysis for studies with different AKI definitions. *CI* confidence interval, *KH* Knapp–Hartung method

Data regarding AKI incidence were available in all 26 studies, and the rate of AKI was significantly lower in the RIPC group than in the control group [p = 0.01; RR 0.79 (95 % CI 0.66–0.95), Fig. 3] (random model). However, it should be noted that different AKI definitions were applied in different studies. The AKI definitions used included the AKI Network (AKIN) criterion [16, 29, 38, 43, 47, 50], the Kidney Disease: Improving Global Outcomes (KDIGO) criterion [14, 41, 48], the RIFLE criterion [39, 46], postoperative serum creatinine ≥0.5 mg/dl or ≥25 % above baseline [30, 34, 37, 40, 45], and others [27, 28, 31–33, 35, 36, 42, 44, 49]. We performed subgroup analyses based on the different AKI definitions. RIPC reduced AKI incidence as defined by a postoperative serum creatinine ≥0.5 mg/dl or ≥25 % above baseline [p = 0.0002; RR 0.42 (0.27–0.67); heterogeneity χ^2^ = 3.89, I^2^ = 0 %, p for heterogeneity = 0.42]. However, RIPC did not reduce AKI incidence in the subgroups in terms of the other AKI definitions: AKIN criterion [p = 0.56; RR 0.87 (0.56–1.37); heterogeneity χ^2^ = 12.22, I^2^ = 59 %, p for heterogeneity = 0.03], KDIGO criterion [p = 0.32; RR 0.83 (0.58–1.20); heterogeneity χ^2^ = 6.76, I^2^ = 70 %, p for heterogeneity = 0.03], RIFLE criterion [p = 0.37; RR 0.87 (0.64–1.18); heterogeneity χ^2^ = 0.06, I^2^ = 0 %, p for heterogeneity = 0.81], and others [p = 0.34; RR 0.83 (0.56–1.22); heterogeneity χ^2^ = 18.85, I^2^ = 52 %, p for heterogeneity = 0.03] (Fig. 4).

#### In-hospital mortality

**Fig. 5:**
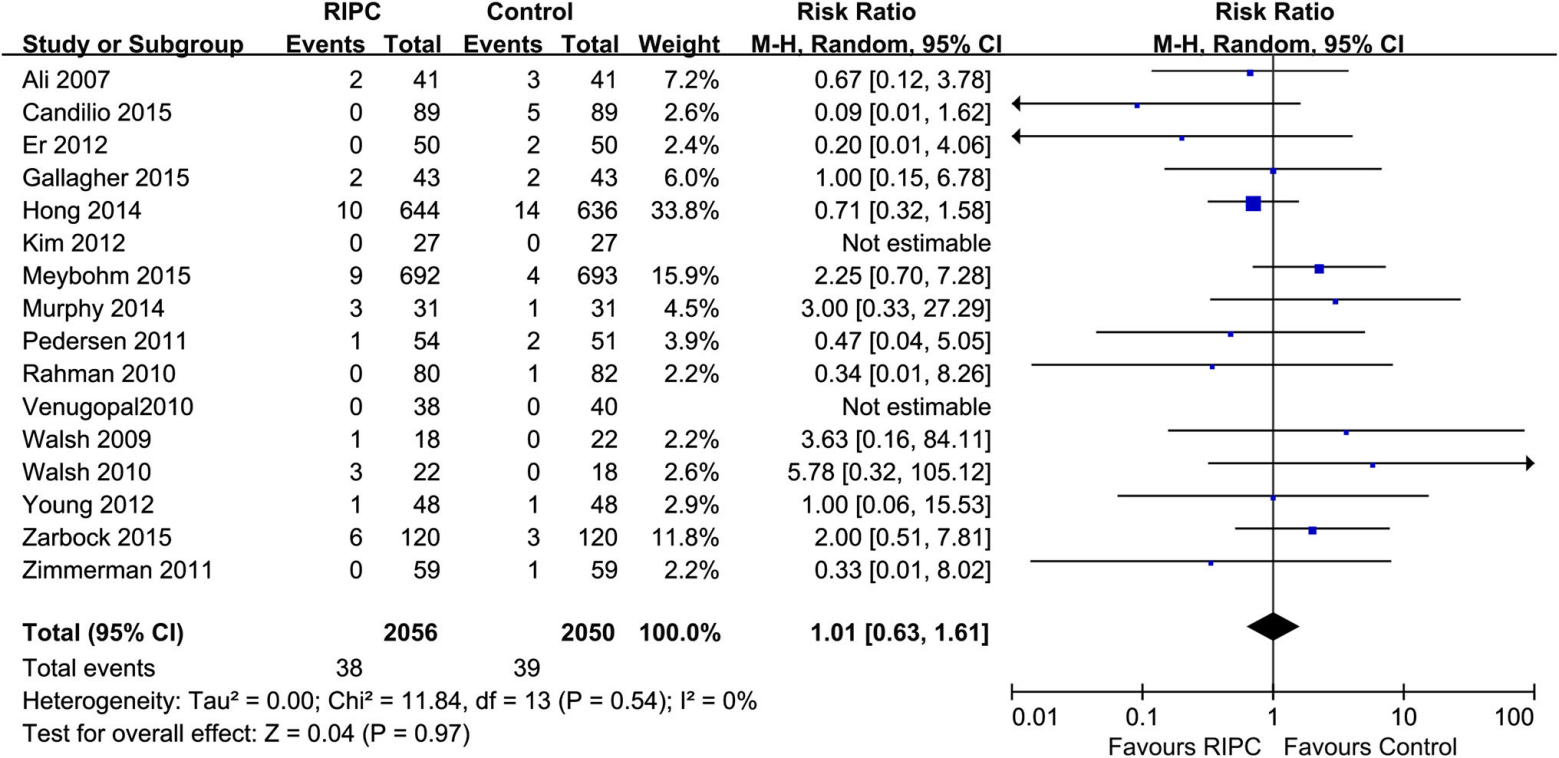
Meta-analysis of in-hospital mortality between RIPC and control groups. *CI* confidence interval, *KH* Knapp–Hartung method

In-hospital mortality was reported in 16 trials [14, 27, 28, 30, 31, 33, 36, 38–40, 42–44, 46, 47, 49], and there was no significant difference in mortality between the RIPC and control groups [p = 0.97; RR 1.01 (0.63–1.61); heterogeneity χ^2^ = 11.84, I^2^ = 0 %, p for heterogeneity = 0.54, Fig. 5].

#### Change in renal biomarkers

**Fig. 6:**
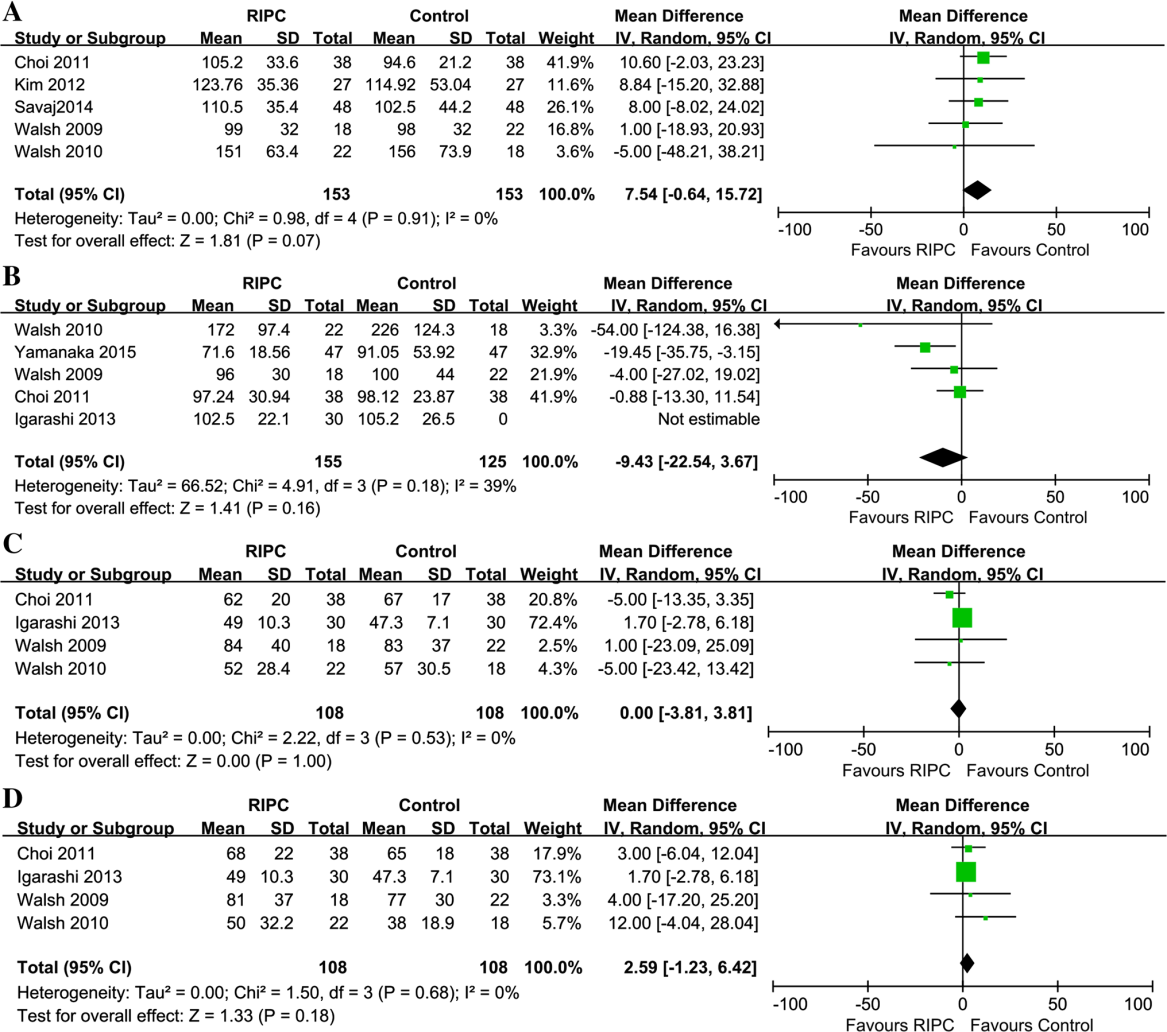
Meta-analysis of mean differences in kidney biomarker levels between RIPC and control groups. **a** Serum creatinine (Scr) levels at 24 h postoperatively; **b** Scr levels at 48 h postoperatively; **c** glomerular filtration rates (GFRs) at 24 h postoperatively; **d** GFRs at 48 h postoperatively. *CI* confidence interval, *KH* Knapp–Hartung method, *SD* standard deviation

At 24 and 48 h after surgery, serum creatinine level was reported in five trials [29, 36, 41, 42, 44] and five trials [29, 35, 42, 44, 45], respectively. GFR was reported at 24 and 48 h after surgery in four trials [29, 35, 42, 44]. There were no significant differences between the two groups in serum creatinine (Scr) level or GFR at 24 or 48 h after surgery (Fig. 6).

#### Initiation of renal replacement therapy

**Fig. 7:**
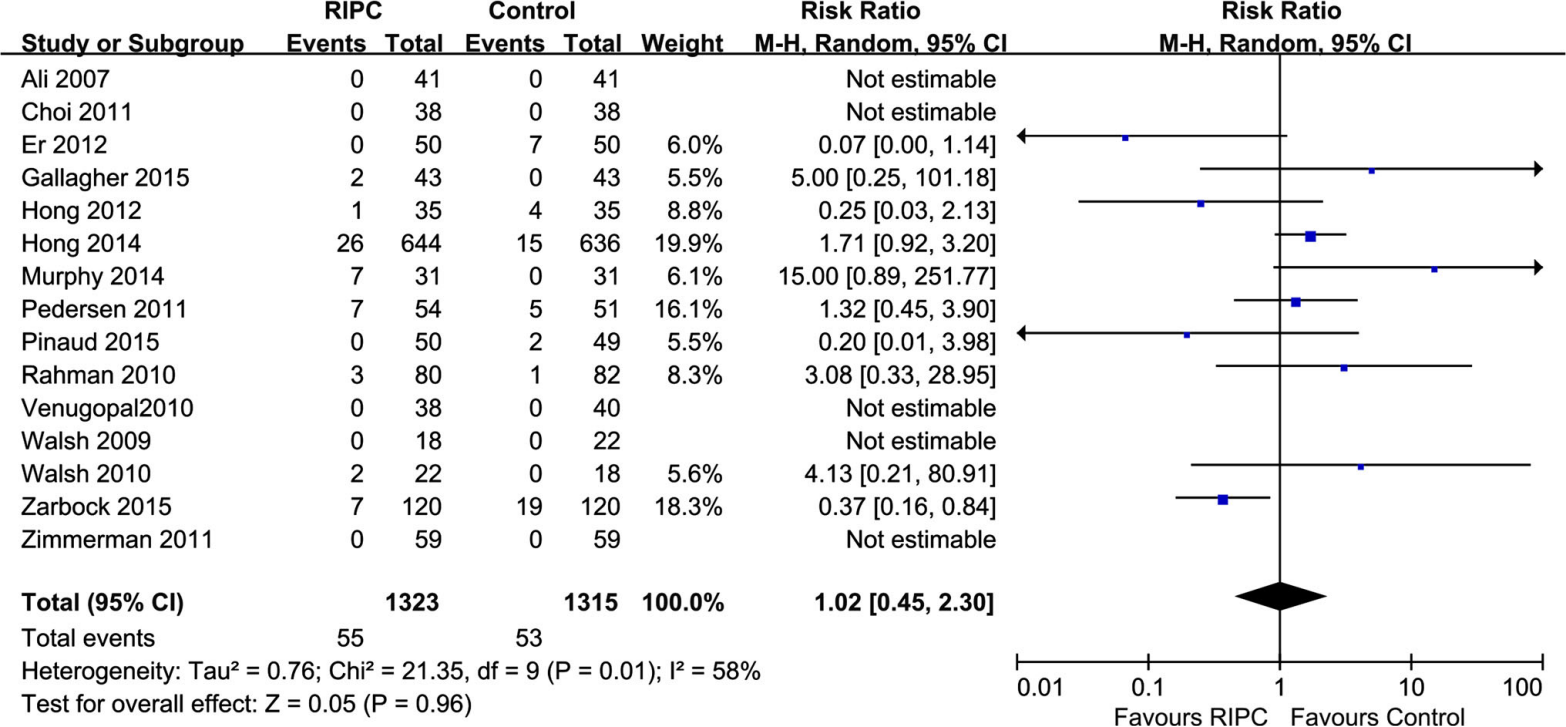
Meta-analysis of the use of renal replacement therapy between RIPC and control groups. *CI* confidence interval, *KH* Knapp–Hartung method

Renal replacement therapy was reported in 15 trials [14, 27, 29–33, 38–40, 42, 44, 47, 50], and no significant difference was observed in the performance of renal replacement therapy between the RIPC group and the control group [p = 0.96; RR 1.02 (0.45–2.30); heterogeneity χ^2^ = 21.35, I^2^ = 58 %, p for heterogeneity = 0.01, Fig. 7].

#### The lengths of hospital stay and ICU stay

**Fig. 8:**
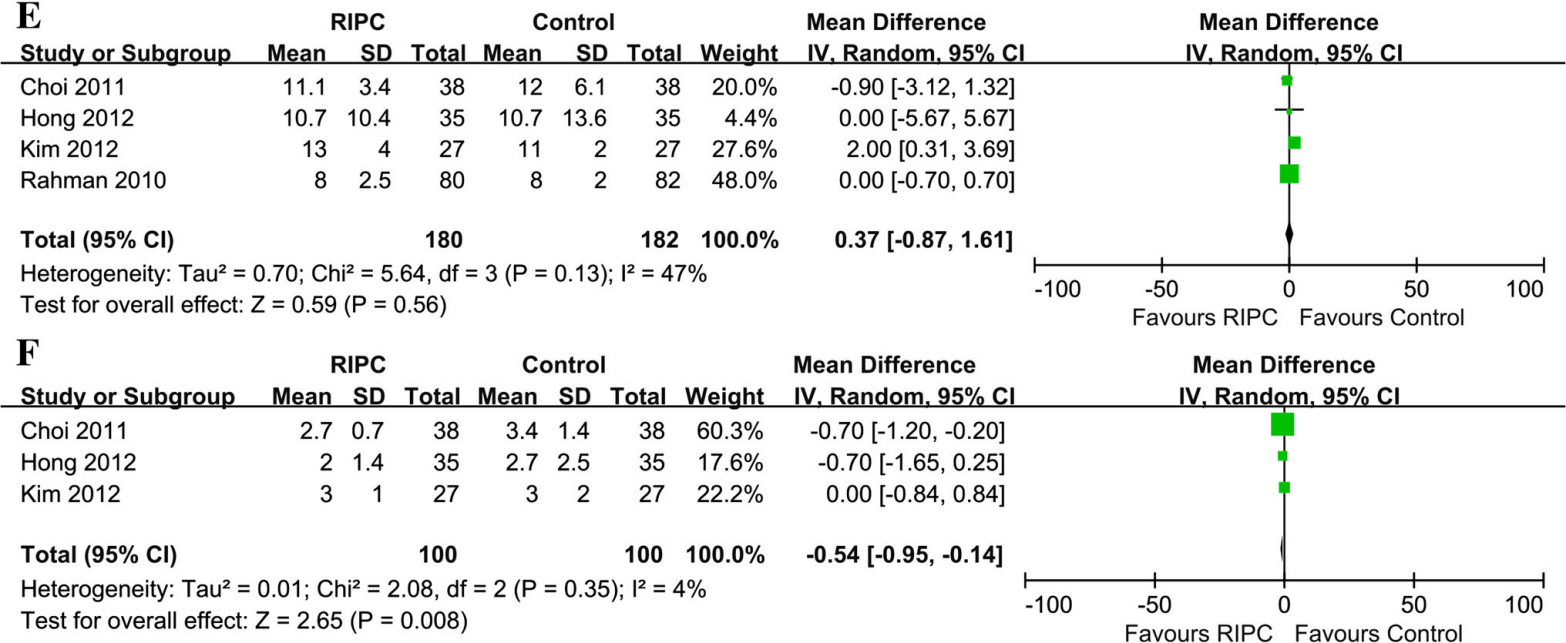
Meta-analysis of **e** length of in-hospital stay and **f** length of intensive care unit (ICU) stay between RIPC and control groups. *CI* confidence interval, *KH* Knapp–Hartung method

Four trials reported the length of hospital stay [29, 32, 36, 40], and three trials reported the length of ICU stay [29, 32, 36]. There was no significant difference in the length of hospital stay between the two groups [p = 0.56; mean difference 0.37 (−0.87 to 1.61); heterogeneity χ^2^ = 5.64, I^2^ = 47 %, p for heterogeneity = 0.13]. The length of ICU stay was remarkably reduced in the RIPC group [p = 0.008; mean difference −0.54 (−0.95 to −0.14); heterogeneity χ^2^ = 2.08, I^2^ = 4 %, p for heterogeneity = 0.35, Fig. 8]; however, the number of trials was too small to observe a statistically significant difference.

#### Subgroup analysis

**Fig. 9:**
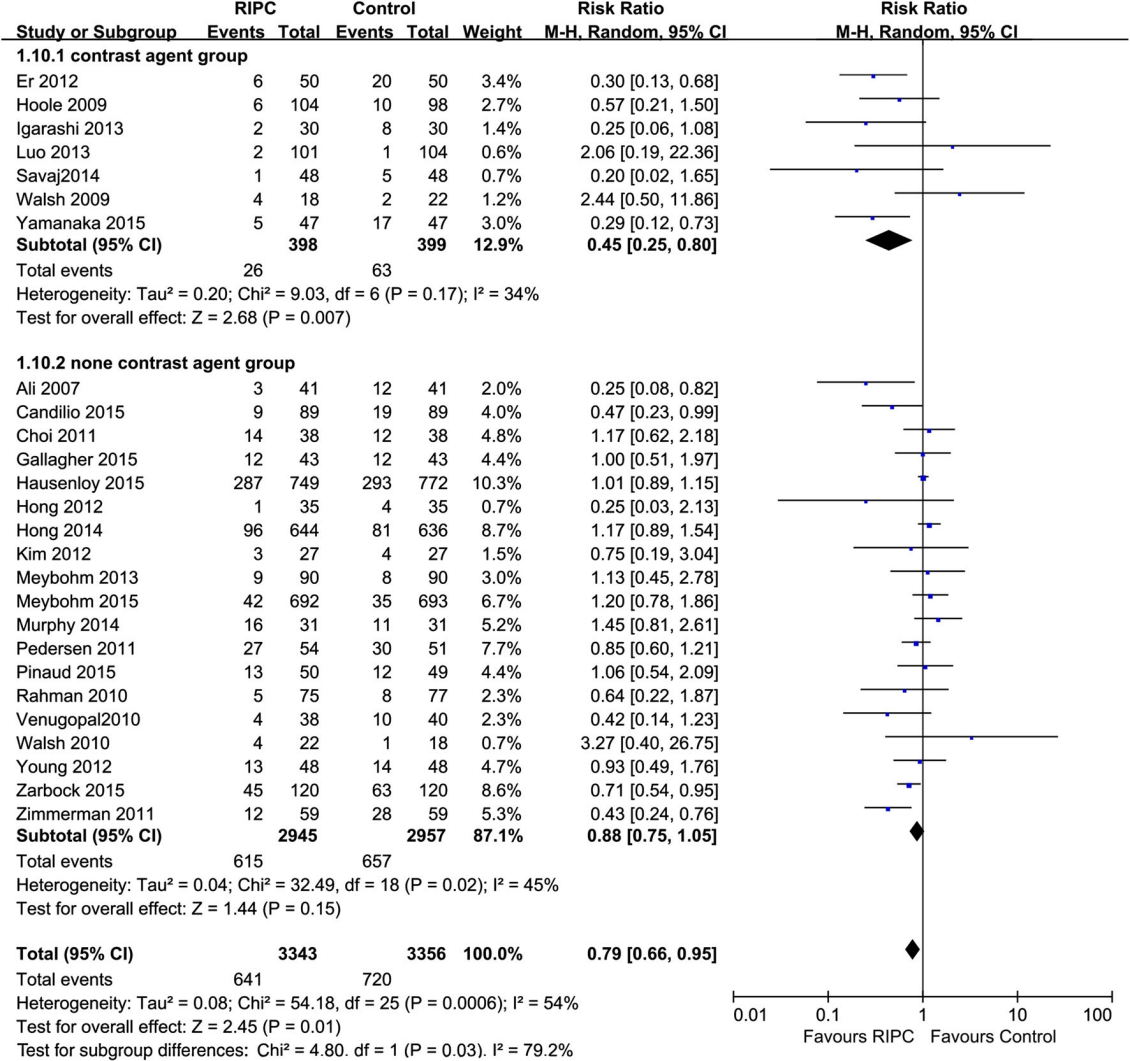
Subgroup analysis based on the use of the contrast medium intervention. *CI* confidence interval, *KH* Knapp–Hartung method

This meta-analysis of AKI incidence showed that RIPC reduces the perioperative incidence of AKI in cardiac and vascular surgery patients. However, there was high statistical heterogeneity among the included trials (heterogeneity χ^2^ = 46.67, I^2^ = 53 %, p for heterogeneity = 0.002, Fig. 3). Since the different AKI definitions are based on different changes in serum creatinine from baseline, and contrast applications are specific clinical settings that could influence kidney function, we performed subgroup analyses of these two potential covariates. The results of the subgroup analysis were marginally significant (Figs. 4, 9); however, meta-regression analysis indicated that different AKI definitions were not the covariate contributing significantly to heterogeneity on the risk estimate for AKI incidence [coefficient −0.39 (−1.15 to 0.38); p = 0.56]. We also did the meta-regression of contrast application conditions, and found that there was statistically significant difference in the risk estimate for AKI incidence [coefficient −0.22 (−0.51 to 0.07); p = 0.039].

### Sensitivity analysis

We used a fixed model and random model to analyze the different outcomes, and none of the results of the examined outcomes were different for these two models. These two models both indicated that remote ischemic preconditioning reduced the incidence of AKI in patients undergoing cardiovascular interventions.

## Discussion

Many randomized controlled trials on remote ischemic preconditioning have been performed, and RIPC has been shown to ameliorate heart ischemia–reperfusion injury [51]. The commonly used RIPC methods are the placement of an inflatable tourniquet around the limbs and the cross-clamping of the iliac arteries, both of which are noninvasive and nonpharmacological procedures. Cardiac and vascular surgery patients have a high risk of AKI [1], and AKI increases mortality [2]. However, currently, there are no effective clinical strategies for preventing the occurrence of AKI [5, 6, 12–14]. Remote ischemic preconditioning is a hot research area, and many researchers have applied this method to prevent AKI in cardiovascular surgery patients in recent years. However, disappointingly, the results of those studies do not clearly show whether RIPC reduces AKI incidence in cardiac and vascular surgery patients. Meta-analyses performed by other teams also failed to reach a consistent conclusion. Yasin et al. [21] and Yang et al. [22] performed meta-analyses, and they both found no statistically significant differences in AKI incidence between cardiovascular surgery patients who did and did not undergo RIPC. However, a meta-analysis of 13 trials conducted by Yang et al. (1134 participants) showed that RIPC decreased the risk of AKI in cardiac and vascular surgery patients [23]. In addition, meta-analyses of other indices of renal impairment have not reached consistent conclusions [19, 21, 22, 52–54]. These inconsistent results may be due to the limitation of small sample size; therefore, larger samples and meta-analyses are needed.

This meta-analysis included 6699 participants in 26 trials who underwent cardiac or vascular interventions and were randomized to a RIPC group or control group. The results of our analysis reveal that RIPC significantly reduced AKI incidence in patients undergoing cardiac or vascular interventions [p = 0.01; RR 0.79 (0.66–0.95)]. Because there was high statistical heterogeneity among the included trials, conclusions based on these results should be made with caution. The meta-analysis by Yang et al. indicated that the contrast medium intervention was not a covariate that significantly contributed to the heterogeneity in the risk estimate for AKI incidence, but the subgroup analysis of the contrast medium intervention in our study showed marginal statistical significance (Fig. 9). So we performed a meta-regression analysis, and the result showed that the contrast medium intervention was a covariate that significantly contributed to heterogeneity in the risk estimate for AKI incidence.

Although AKI incidence was reduced in the RIPC group, there were no significant differences in mortality or renal biomarkers between the two groups. Considering that not all of the included trials reported mortality or renal biomarkers, it is difficult to confirm whether RIPC has a kidney protective effect in patients undergoing cardiovascular interventions. Furthermore, other more sensitive indicators of early kidney damage, such as neutrophil gelatinase-associated lipocalin (NGAL) [55], Cys C [56] and urine output, were not available. Likewise, the use of renal replacement therapy and the length of hospital stay were not significantly different between the two groups. Length of ICU stay was shorter in the RIPC group, but that finding cannot be considered conclusive because only three trials reported valid data regarding ICU stay.

One trial only included patients without diabetes mellitus (DM) [43], whereas another trial only included patients with DM [41], and as DM is a potential risk factor for postoperative acute kidney injury in patients undergoing cardiac and vascular surgeries [57] the findings of our study may not be generalizable to non-diabetic patients.

Our meta-analysis has some limitations. First, surgery type, anesthesia and premedication varied between trials, which may have generated different risk levels of perioperative acute kidney injury incidence. Second, there was a high level of heterogeneity in the demographic data of the patients among the included trials: Also, the baseline serum creatinine level varied considerably between studies, which may indicate differences in basic renal function between studies. We should also note that the patients in the trial by Pedersen et al. [39] were children, while those in the other 25 studies were adults. Third, we did not limit this meta-analysis to studies that examined one specific RIPC procedure: two of the included studies performed cross-clamping of the iliac arteries [27, 44] instead of using an inflatable tourniquet around the limbs. Fourth, different AKI definitions were applied by the researchers of the different studies. All of these limitations may explain the high heterogeneity between studies besides the contrast application. Finally, only ten studies [14, 16, 28, 33, 36, 38, 40, 45, 46, 49] were double-blind, and we think that the single-blind and non-blind studies may have influenced the results of this meta-analysis.

## Conclusion

Remote ischemic preconditioning can reduce the postoperative occurrence of acute kidney injury in cardiac and vascular surgery patients. However, considering the high heterogeneity among the 26 trials analyzed, we cannot draw a definitive conclusion regarding the value of RIPC at this time. A larger sample using a uniform AKI definition and RIPC method is needed to reach a more definitive conclusion.
